# Exercise and Fatty Heart

**Published:** 1888-11

**Authors:** 


### EDITORIAL.

### EXERCISE AND FATTY HEART.

Recently there has been quite a revival of the suggestion, made many years ago by Professor Stokes of Dublin, of the advantage resulting in fatty heart from systematic muscular exercise, as by gymnastics, mountain climbing, etc.

Doubtless, within certain limits, this advice may be followed with advantage, either with or without medication; but probably the greatest danger attending such efforts lies in the difficulty of securing moderation, and progressive increase in the amount of the exercise,without which danger to life would be materially augmented. Similar debility of the recti muscles is frequently found in cases of asthenopia, and is benefited by systematic and progressive exercise of those muscles. Both instances are but illustrations of what has been so long and so well known of not only the advantage but the necessity of proper muscular exertion, confined within the recuperative power of the muscles exercised. It seems requisite that old truths should often be retold, in new ways and with new illustrations, that sight of them shall not be lost. At present this is one of the illustrations, with a useful and practical object in view. It is to be commended if confined to the limits of safety, but the dangers, as well as the advantages, should be fully presented, with reliable guides to the recognition of impending danger, and a safe course out of it. Without these there will doubtless be other victims added to the list of those who have spasmodically evinced more enthusiasm than discretion in the matter of physical exercise, especially in the now popular athletics.

